# Effect of Energy Provision Strategy on Rumen Fermentation Characteristics, Bacterial Diversity and Community Composition

**DOI:** 10.3390/bioengineering10010107

**Published:** 2023-01-12

**Authors:** Qinghua Qiu, Jiantong Zhang, Mingren Qu, Yanjiao Li, Xianghui Zhao, Kehui Ouyang

**Affiliations:** Jiangxi Province Key Laboratory of Animal Nutrition/Engineering Research Center of Feed Development, Institute of South Grassland Research, Animal Nutrition and Feed Safety Innovation Team, College of Animal Science and Technology, Jiangxi Agricultural University, Nanchang 330045, China

**Keywords:** bacterial community composition, diet shift, energy provision strategy, rumen bacterial diversity, rumen fermentation characteristic

## Abstract

This study was conducted to explore the rumen fermentation characteristics, bacterial diversity, and community composition of Hu sheep under four energy provision strategies. Ninety-six Hu sheep (body weight: 17.78 ± 1.24 kg) were equally assigned to four energy provision strategies: (1) low-energy diet for the whole finishing stage (LL); (2) high-energy diet for the whole finishing stage (HH); (3) low-energy diet in the early finishing stage and high-energy diet in the late finishing stage (LH); (4) high-energy diet in the early finishing stage and low-energy diet in the late finishing stage (HL). The results showed that the proportion of acetate was lower in the HH group than that in the HL group, whereas the opposite result was observed for the butyrate proportion (*p* < 0.05). The Chao 1, observed species, PD whole tree, and Shannon index of the rumen bacteria were higher in the LL group than that in the HH group (*p* < 0.05). The taxonomic annotations revealed that the Patescibacteria, *Rikenellaceae RC9 gut group*, *Christensenellaceae R-7 group*, and *Anaeroplasma* abundances were higher in the HL group than that in the HH group, and the opposite results were observed regarding the relative abundances of *Selenomonas* and *Anaerovibrio* (*p* < 0.05). The relative abundances of Spirochaetota and *Treponema* were higher in the LH group than that in the HH group (*p* < 0.05). Moreover, the analysis of similarity (ANOSIM) showed significant differences between groups (R = 0.6792 and *p* = 0.001). This study indicates that the energy provision strategy had little impact on the rumen fermentation characteristics, while it heavily affected the rumen bacterial diversity and community composition. This study may provide insight into the rumen fermentation characteristics and bacterial community under routine finishing models and contribute to the optimization of energy provision strategies of Hu sheep.

## 1. Introduction

Ruminal microbes allow ruminants to convert plant materials and nonprotein nitrogen into highly available nutrients to meet their requirements of body maintenance and product output. As the most diverse microbial community and the largest component of the microbial biomass in ruminal microbes, rumen bacteria have been extensively explored in recent years [1,2,3]. The structural composition of the rumen bacterial community is affected by a great number of internal and external factors, such as host, physiological status, diet, and environment [4]. For instance, a high nutrient density diet decreased the rumen bacterial diversity and increased the relative abundances of bacteria participating in amylolysis [5], while a greater forage intake increased the alpha diversity and showed substantially different bacterial communities when compared to sheep fed a concentrate-rich diet [6]. Numerous studies have revealed the vast interactions between rumen bacteria and animal efficiency and performance [1,7]. Therefore, rumen bacteria are potentially intermediate bridges to uncover the internal mechanism of diet provision on an animal’s productive performance and could further provide decision making for ruminants production.

Ruminants obtain energy from degrading dietary nutrients to maintain their vital functions and to produce palatable food for humans. An optimized dietary energy provision strategy, for instance, the amount and supply order across each finishing stage, plays decisive roles in improving the feed efficiency, economic benefit, and sustainability of ruminant production. Generally, three popular feeding strategies are adopted in practical Chinese sheep production; the first two are high- or low-density energy feeding in the whole finishing stage, and the last strategy is divided into two stages, with low-density energy for the early finishing stage and high-density energy for the late finishing stage [8]. However, the energy density in the late finishing stage may be shifted to low density due to the fact of an insufficient grain supply or pessimistic market quotation. Previous studies revealed that continuous high-density feeding could achieve more weight gain and a higher average daily gain; meanwhile, unfavorable effects were observed in rumen fermentation characteristics and bacterial diversity [5,9]. Rojen et al. [10] found a compensate effect on cows fed a low-nitrogen diet, with a greater capacity to recycle urea nitrogen. Qiu et al. [5] revealed carry-over effects on nutrient digestibility and bacterial diversity when the diet shifted. These findings indicate that a diet shift may influence the final consequences due to the fact of compensate or carry-over effects. Many studies have investigated the impacts of a constant high- or low-energy feeding regime on rumen fermentation characteristics and bacterial community [11,12,13,14,15]. These investigations have provided valuable insights into optimizing a feeding strategy for practical livestock production. However, information on the other two energy provision strategies with a diet shift (i.e., low to high and high to low) is limited. Therefore, it is still necessary to explore the rumen fermentation characteristics and bacterial community under these energy provision strategies to evaluate their practical feasibility.

This study aimed to explore the effects of four energy provision strategies on rumen fermentation characteristics, rumen bacterial diversity and community composition. We hypothesized the energy provision strategies with a diet shift would generate distinguishable results from that of continuous high- or low-energy provision.

## 2. Materials and Methods

### 2.1. Animals and Experimental Design

**Figure 1:**
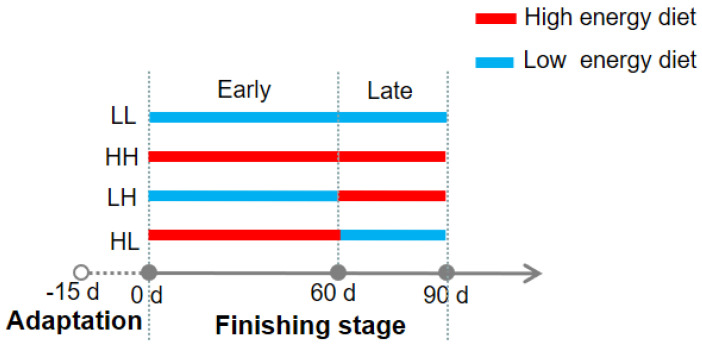
Energy supply order for each provision strategy.

Animal care and welfare guidelines were carried out with the authorization of the Committee for the Care and Use of Experimental Animals at Jiangxi Agricultural University under protocol number JXAULL-2021036. A total of ninety-six Hu sheep, with an average weight of 17.78 ± 1.24 kg, were selected to conduct the current study. The animals were equally assigned to four energy provision strategies: (1) low-energy diet for the whole finishing stage (LL); (2) high-energy diet for the whole finishing stage (HH); (3) low-energy diet in the early finishing stage and high-energy diet in the late finishing stage (LH); (4) high-energy diet in the early finishing stage and low-energy diet in the late finishing stage (HL). The early finishing stage lasted for 60 d, and the late finishing stage lasted for 30 d. An illustration of the energy supply order for each provision strategy is shown in Figure 1. The metabolizable energy of the high- and low-energy diets were 2.59 and 2.11 Mcal/kg, respectively. Both of these energy designs were according to the Nutrient Requirements of Meat-Type Sheep and Goat [8], and the detailed ingredient and chemical composition of the high- and low-energy diets are listed in Table 1. The animals were fed in 24 pens, with four sheep in each pen and six pens in each provision strategy. The feeds were provided at 8:00 and 17:00 each day, and approximately 10% of the feed residues was controlled to allow for *ad libitum* feeding. All animals had free access to clean water. The whole trial lasted for 105 d, during which 15 d were designated as an adaptation period and 90 d comprised the experimental period.

### 2.2. Sample Collection

At the end of the experiment, twenty-four sheep were randomly selected from each pen for slaughter after a 12 h fast. The rumen contents were obtained from the dorsal, central, and ventral sites of the rumen after the rumen was separated at the slaughterhouse. The rumen pH value was immediately measured by a portable pH meter (Testo 206, Testo AG, Schwarzwald, Germany). The rumen contents were filtered by four layers of gauze to obtain rumen fluid and preserved at −80 °C for one month, which was the sample used for the rumen fermentation characteristics determination and DNA extraction.

### 2.3. Rumen Fermentation Characteristics Determination

The rumen fermentation characteristics determined in this study included the pH value, ammoniacal nitrogen (NH_3_-N), microbial crude protein (MCP), and volatile fatty acids (VFAs). The concentration of NH_3_-N was determined according to Broderick and Kang [16], wherein the phenol-hypochlorite colorimetric method was used. The MCP concentration was determined by the method of Folin phenol based on Lowry’s assay, as described in Makkar et al. [17]. The individual VFAs, including acetate, propionate, isobutyrate, butyrate, isovalerate, and valerate, were measured using a gas chromatograph (GC-2014 Shimadzu Corporation, Kyoto, Japan). The injection parameter and oven procedure were according to Wei et al. [18]. Branched chain volatile fatty acids (BCVFAs) were calculated as the sum of isobutyrate, valerate, and isovalerate. Rumen fermentation patterns were reflected by the acetate-to-propionate ratio, nonglucogenic-to-glucogenic-acids ratio (NGR), and fermentation efficiency (FE). The NGR and FE were calculated according to Wang et al. [19], as Equations (1) and (2), respectively.

NGR = (C2 + 2 × C4 + C5)/(C3 + C5)(1)

FE = (0.622 × C2 + 1.092 × C3 + 1.56 × C4)/(C2 + C3 + 2 × C4)(2)

In Equations (1) and (2), C2, C3, and C4 indicate acetate, propionate, and butyrate, respectively. In Equation (1), C5 indicates valerate.

### 2.4. DNA Extraction, Sequencing, and Data Analysis

A total of twenty-four rumen fluid samples were used for the DNA extraction with a corresponding commercial kit (OMEGA, Omega Bio-Tek, Norcross, GA, USA), with strict execution as per the manufacturer’s guidelines. The purity and quality of the extracted DNA were assessed on 1% agarose gel and an ultrafine spectrophotometer (NanoDrop 2000, Thermo Fisher Scientific, Waltham, MA, USA), respectively. The bacterial V3 to V4 regions were chosen as the target gene fragment, and they were amplified using the same primers as described in Wei et al. [18]. The subsequent amplification system and program were according to our previous study [5], with three repetitions for each sample. The PCR products were checked on 1% agarose gels and purified by an AxyPrep DNA Gel Extraction kit (Axygen Biosciences, Union City, CA USA). The purified PCR products were delivered to Allwegene Gene Technology Co., Ltd., (Nanjing, China) for high throughput sequencing using an Illumina Miseq PE300 platform (San Diego, CA USA), wherein the paired-end reads were obtained for the subsequent data analysis.

The sequencing data were analyzed using the quantitative insights into microbial ecology (QIIME 2, https://qiime2.org/, accessed on 10 November 2022; [20]). The paired end reads were merged by the Fast Length Adjustment of Short reads (FLASH, version 1.2.11, http://ccb.jhu.edu/software/FLASH/, accessed on 10 November 2022; [21]), which allowed a maximum mismatch rate of 0.10 and a minimum overlap of 10 bp. Sequences were removed if their length was not between 200 and 500 bp, quality score was less than 20, contained ambiguous bases or chimeric sequences, or were mismatched to primer sequences or barcode tags. The qualified sequences were denoised into amplicon sequence variants (ASVs) using the Deblur algorithm of QIIME 2. The ASVs across the samples were rarefied to the lowest sample depth of 48,141 reads, with an average number of ASVs of 653. The taxonomic classifications for each ASV were performed by means of the Ribosomal Database Project (RDP) Classifier, with a confidence threshold of 70% and assigned against the bacterial SILVA 132 database. Five alpha diversity metrics, including Chao 1, observed species, phylogenetic diversity (PD) whole tree, Shannon index, and Simpson index, were introduced to show the richness and evenness of the rumen bacteria using QIIME 2. To better demonstrate the differences and similarities among the four energy provision strategies, principal coordinates analysis (PCoA) and nonmetric multidimensional scaling (NMDS) were conducted using R software (version 4.0.2) based on Bray–Curtis distances. An analysis of similarity (ANOSIM) was performed to uncover the similarities among four energy provision strategies using the vegan package in the R software. An LDA effect size (LEfSe) analysis, including LDA value distribution histogram and cladogram, was performed to show the biomarkers in each provision strategy using LEfSe software based on an LDA score threshold of 4.0 [22].

### 2.5. Statistical Analysis

The MIX procedure of SPSS (version 20; IBM Corporation, Armonk, NY, USA) was used to analyze the data with the following model: Y*_ij_* = *µ* + S*_i_* + R*_j_* + e*_ij_*, where Y*_ij_* is the dependent variable, *µ* is the overall mean, S*_i_* is the fixed effect of the energy provision strategy, R*_j_* is the random effect of the sheep, and e*_ij_* is the residual effect. *Post hoc* multiple comparisons for the different energy provision strategies were performed using Tukey’s test, and the significance was declared at 0.05 (*p* < 0.05).

## 3. Results

### 3.1. Rumen Fermentation Characteristics

The effect of the energy provision strategy on the rumen fermentation characteristics is shown in Table 2. No significant differences were observed in the pH value, concentrations of NH_3_-N, MCP, total VFAs, individual VFA, and rumen fermentation patterns among the groups (*p* > 0.05). The proportion of acetate was higher in the HL group than that in the HH group, whereas the HH group showed a higher value than the HL group regarding the butyrate proportion (*p* < 0.05).

### 3.2. Alpha-Diversity Metrics of Rumen Bacteria

The effect of the energy provision strategy on the rumen bacterial alpha-diversity is listed in Table 3. The Chao 1, observed species, and PD whole tree were higher in the LL group than that in the other three groups, and the Shannon index of the LL group was higher than that in the HH group (*p* < 0.05). However, no significant differences were observed in the Simpson index among the groups (*p* > 0.05).

### 3.3. Rumen Bacterial Community Composition

As shown in Table 4, ten phyla were annotated with a relative abundance greater than 0.1%, with Bacteroidota and Firmicutes dominating with 63.16% and 32.34%, respectively. The relative abundance of Spirochaetota was higher in the LH group than that in the LL and HH groups, and the Patescibacteria abundance was higher in the HL group than that in the HH group (*p* < 0.05).

The taxonomic analysis at the level of genus revealed twenty genera with a relative abundance above 0.5% (Table 5). The highest relative abundance genera were *Prevotella* (37.73%) and *Rikenellaceae RC9 gut group* (9.32%), and the latter showed a higher abundance in the HL group than that in the HH group (*p* < 0.05). The relative abundances of *Christensenellaceae R-7 group* and *Anaeroplasma* were higher in the HL group than that in the other three groups. The *Selenomonas* and *Anaerovibrio* abundances were higher in the HH group than that in the HL group. The *Treponema* showed a higher relative abundance in the LH group than that in the LL and HH groups.

### 3.4. Beta-Diversity of Rumen Bacteria

**Figure 2:**
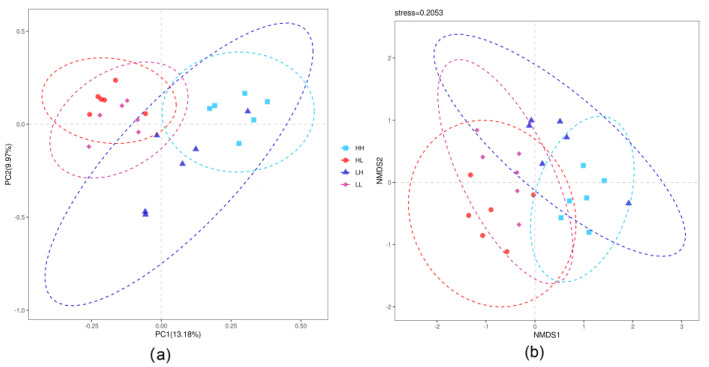
Beta-diversity of the rumen bacteria under four energy provision strategies: (**a**) principal coordinates analysis (PCoA); (**b**) nonmetric multidimensional scaling (NMDS).

As shown in Figure 2, part intersections were observed between the LL and HL groups and between the LH and HH groups. The ANOSIM showed significant differences between groups (R = 0.6792 and *p* = 0.001).

### 3.5. Biomarker Analysis

**Figure 3:**
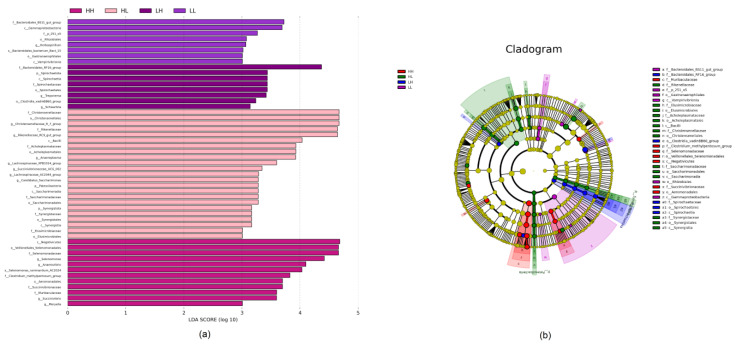
Effect of the energy provision strategy on the discriminative bacterial communities at different taxonomic levels: (**a**) linear discriminant analysis (LDA) score generated from the LEfSe analysis; (**b**) cladogram derived from the LEfSe analysis.

A biomarker analysis was performed to reveal the differential species among the groups at different taxonomic levels, presented in the form of an LefSe (LDA distribution bar graph, Figure 3a) and cladogram (Figure 3b). The LDA distribution bar graph shows that a total of thirteen species were observed with an LDA score greater than 4.0 and a significance of *p* < 0.05. The biomarkers with significant discriminative power were Bacteroidales RF16 group in the LH group; Christensenellaceae, Christensenellales, *Christensenellaceae R-7 group*, Rikenellaceae, *Rikenellaceae RC9 gut group*, and Bacilli in the HL group; and Negativicutes, Veillonellales Selenomonadales, Selenomonadaceae, *Selenomonas*, *Anaerovibrio*, and *Selenomonas ruminantium AC2024* in the HH group.

## 4. Discussion

The energy provision strategy did not affect the rumen fermentation characteristics, except for the proportions of acetate and butyrate. The concentration of ruminal NH_3_-N is associated with dietary protein degradation, and more dietary protein content yields more NH_3_-N [23]. In this study, no differences were observed among the four energy provision strategies due to the similar crude protein levels in the high- and low-energy diet (15.83% vs. 15.76%). It was reported that the MCP production was affected by degraded protein utilization, microbial composition, and activity [24]. The current study showed no significant differences among the groups, probably due to the similar degraded protein, which could be indirectly verified by the similar NH_3_-N concentrations. VFAs are known to be the main form of energy utilization for ruminants, and their concentrations depend on the dietary composition, carbohydrate digestion, microbial species, and VFA absorption rates [25]. It is interesting to see the numerically higher total VFA concentration in the LL group than the HH group; a similar phenomenon was also reported by Wang et al. [11], and the current results may be due to the fact that the VFA absorption rate of the high-grain diet was quicker than the high-fiber diet [26]. The fermentation of structural carbohydrates produced more acetate than the fermentation of starch [25], which well explains the higher proportion of acetate in the HL group than the HH group due to the higher amount of roughage in the low-energy diet. Dijkstra et al. [25] reported that high substrate concentrations shifted the acetate fermentation pattern to a butyrate fermentation pattern, and the butyrate molar proportion increased when bacteria were exposed to a low pH environment, which provides evidence for the present result of a higher butyrate proportion in the HH group.

High-energy provision strategy reduced the richness and evenness of rumen bacteria diversity, which were indicated by the Chao 1, observed species, PD whole tree, and Shannon index. Previous studies revealed that a high nutrient density diet or grain-based diet decreased the ruminal alpha diversity [5,27]. The current study showed a similar phenomenon; this result could be explained by the numerical pH value reduction in the HH group, because a low ruminal pH would decrease the bacterial diversity [28]. Interestingly, marked differences were observed between the LL and HL groups in the Chao 1, observed species, and PD whole tree, even though the same energy diet was provided for them in the late finishing stage, indicating that the impact of high-energy feeding could not be diluted by one month of low-energy feeding. These results may provide insight into the strategy for the alteration of the plasticity on the bacterial diversity from the perspective of the diet model, as well as attract attention to the carry-over effects on rumen bacterial diversity.

Taxonomic annotations provide deep insight into the rumen bacterial abundance at the phylum and genus levels under different energy provision strategies. Spirochaetota was reported to be involved in degrading carbohydrates [29]; thus, similar abundances were expected between the LL and HH groups. However, the LH group showed a higher relative abundance of this phylum than the LL group, indicating that a dietary energy shift may trigger a compensatory degradation of organic matter (85.0% vs. 83.6% in the LH and LL groups, respectively), and this explanation could be further verified from a similar abundance between the LH and HL groups. Patescibacteria is the dominant member of the groundwater microbiome [30] and shows great potential to adapt to an adverse environment [31]. Therefore, it is reasonable to see a higher abundance of this phylum in the HL or LH group than the LL or HH group due to the diet shift after the early finishing stage. The *Rikenellaceae RC9 gut group* was reported to play a vital role in degrading fiber [5]. In this study, a higher relative abundance of this genus was observed in the HL group than the HH group, which resembled that reported by Qiu et al. [5], who found a lower abundance of it in the rumen of cattle fed a high-density diet. The family Christensenellaceae played a vital role in fermenting fiber and starch into acetate and butyrate [32], and bacteria from *Christensenellaceae R-7 group* were found to be beneficial to the body’s health [33,34]. It is reasonable to see a higher relative abundance of *Christensenellaceae R-7 group* in the HL group than the HH group, because a previous study revealed that a high-density diet had negative impacts on serum metabolism and visceral morphology [9]. As mentioned above, as a genus belonging to Christensenellaceae, *Christensenellaceae R-7 group* may also be involved in the production of acetate, which is in line with the higher proportion of acetate in the HL group. Similarly, the genus *Anaeroplasma*, an obligate anaerobe, was reported to be an indicator of a healthier status and was found to have a higher abundance in lambs fed more roughage [35]. Our results also showed a higher abundance of *Anaeroplasma* in the HL group than the HH group, because the low-energy density diet contained more roughage. *Treponema* was previously regarded as a ruminal microbe involved in cellulose degradation [36] and in fructose polymers digestion and utilization [37]. However, a low abundance of *Treponema* was observed in both the LL and HH groups, while the LH and HL groups showed high abundances, indicating that a diet shift may favor the growth of *Treponema*, and the shift from high fiber to high starch may expedite the process. *Selenomonas* is a widespread rumen bacteria that possesses the capability of degrading both starch and cellulose [36], and the maximal growth of *Selenomonas ruminantium* was achieved under sufficient energy media [38]. Therefore, it is easy to expect the current higher abundance of *Selenomonas* in the HH group because of the greater dietary energy provision. The increase in the *Anaerovibrio* abundance in the HH group may be the cause of the higher ether extract in the high-energy diet, because *Anaerovibrio* was reported to be the core rumen bacteria in degrading fat [36,39].

The biomarker analysis revealed more discriminative taxa at the levels of phylum, class, order, family, genus, and species. Except for the aforementioned phyla and genera, as well as their subordination of class, order, family, and species, the classes of Bacilli and Negativicutes and the family of Bacteroidales RF16 group were biomarkers in the HL, HH, and LH groups, respectively. The class of Bacilli were distributed in the feed particles and greatly contributed to the breakdown of plant polysaccharides [40]. Furthermore, Nardi et al. [41] reported that the relative abundance of Bacilli was easily affected when they underwent diet adaptation. The present study also revealed a higher relative abundance of Bacilli in the HL group, which is consistent with the above findings. The class of Negativicutes was previously classified as Clostridia and was recently categorized as a new class with a division of three orders: Selenomonadales, Veillonellales, and Acidaminococcales [42]. Zhang et al. [43] found a higher abundance of Negativicutes in the rumen of sheep with a high residual feed intake, as well as a positive association between Selenomonadales and residual feed intake; this class may have potential roles in nutrient supply. The HH group showed a higher abundance of Negativicutes, suggesting the nutrient utilization efficiency of this energy provision strategy may be low. Bacteroidales RF16 group is a potential producer of VFAs and plays an active role in maintaining the gut homeostasis; furthermore, its abundance is sensitive to heat stress or acid stress induced by a diet shift [44,45]. A higher abundance of Bacteroidales RF16 group was observed in the LH group, probably due to the diet shift from low-energy density to high-energy density. Its incremental abundance may suggest that the optimized energy provision strategy would be low energy for the early finishing stage and high energy for the late finishing stage, which coincides well with the energy provision recommendation of the popular feeding standard [8].

## 5. Conclusions

Taken together, the energy provision strategy did not affect the rumen fermentation characteristics, except for the proportions of acetate and butyrate. The strategy of continuous high-energy provision (HH) decreased the rumen bacterial richness and evenness and altered the relative abundances of part of the bacteria. These strategies with a diet shift from high- to low-energy provision (HL) or from low- to high-energy provision (LH) increased the relative abundances of Spirochaetota, Patescibacteria, *Rikenellaceae RC9 gut group*, *Christensenellaceae R-7 group*, *Anaeroplasma*, and *Treponema*. This study profiled the rumen fermentation characteristics, bacterial diversity, and community composition under four energy provision strategies and may provide insight into discriminative bacteria when a diet shifts.

## Figures and Tables

**Table 1:** Ingredient and chemical composition of the low- and high-energy diets.

Item	Low-Energy Diet	High-Energy Diet
Ingredient		
Corn, g/kg	111.4	490.9
Wheat bran, g/kg	46.2	18.1
Soybean meal, g/kg	110.9	143.1
Peanut straw, g/kg	687.9	304.1
Calcium hydrophosphate, g/kg	1.5	1.5
Sodium bicarbonate, g/kg	2.4	2.5
Salt, g/kg	4.9	5.0
Premix ^1^, g/kg	34.8	34.8
Total	1000	1000
Chemical Composition		
Metabolizable energy, Mcal/kg	2.11	2.59
Crude protein, g/kg	157.6	158.3
Ether extract, g/kg	92.8	111.6
Neutral detergent fiber, g/kg	435.2	280.4
Acid detergent fiber, g/kg	322.8	158.6

^1^ The provided premix per kg: 250 mg of Cu; 1400 mg of Fe; 900 mg of Mn; 1200 mg of Zn; 100,000 IU of VA; 27,000 IU of VD3; and 800 IU of VE.

**Table 2:** Effect of the energy provision strategy on the rumen fermentation characteristics of Hu sheep.

Item ^1^	LL ^2^	LH ^3^	HL ^4^	HH ^5^	SEM ^6^	*p*-Value
pH value	7.04	7.36	7.24	6.80	0.244	0.415
Ammoniacal nitrogen, mg/dL	15.85	16.00	12.00	15.09	1.462	0.213
Microbial crude protein, mg/L	526.13	454.87	624.08	703.97	80.88	0.175
Total volatile fatty acids, mM	67.52	44.89	51.79	54.79	10.75	0.521
Concentration, mM						
Acetate	46.42	30.69	37.08	33.61	6.994	0.433
Propionate	11.24	7.28	7.38	10.07	2.722	0.668
Isobutyrate	0.48	0.36	0.48	0.45	0.085	0.735
Butyrate	8.03	5.46	5.55	9.33	1.801	0.364
Isovalerate	0.60	0.51	0.67	0.58	0.089	0.640
Valerate	0.75	0.57	0.63	0.75	0.107	0.597
BCVFAs	1.82	1.45	1.79	1.77	0.244	0.679
Proportion, %						
Acetate	69.82 ab	68.68 ab	71.16 a	62.97 b	1.878	0.029
Propionate	16.32	16.50	14.78	16.77	2.337	0.931
Isobutyrate	0.89	0.87	0.96	0.86	0.128	0.947
Butyrate	10.63 b	11.44 b	10.40 b	16.93 a	1.332	0.007
Isovalerate	1.10	1.17	1.41	1.11	0.181	0.597
Valerate	1.23	1.34	1.30	1.36	0.111	0.845
BCVFAs	3.22	3.38	3.66	3.32	0.382	0.865
Rumen Fermentation Pattern						
Acetate-to-propionate ratio	4.48	4.21	4.10	4.41	0.430	0.919
NGR	5.52	5.29	4.90	6.30	0.630	0.484
Fermentation efficiency	0.72	0.73	0.73	0.74	0.008	0.579

^1^ BCVFAs, branched chain volatile fatty acids—the sum of isobutyrate, isovalerate, and valerate. NGR, nonglucogenic-to-glucogenic-acids ratio. ^2^ LL denotes the low-energy diet for the whole finishing stage. ^3^ LH denotes the low-energy diet in the early finishing stage and high-energy diet in the late finishing stage. ^4^ HL denotes the high-energy diet in the early finishing stage and low-energy diet in the late finishing stage. ^5^ HH denotes the high-energy diet for the whole finishing stage. ^6^ SEM, standard error of the mean. Different lowercase letters (“a” or “b”) within the same row indicate differences, “ab” indicates both similarities with “a” and “b”.

**Table 3:** Effect of the energy provision strategy on the rumen bacterial alpha-diversity metrics of Hu sheep.

Item	LL	LH	HL	HH	SEM	*p*-Value
Chao 1	1060.80 a	572.81 b	567.39 b	499.13 b	74.15	<0.001
Observed species	972.58 a	557.00 b	557.00 b	481.15 b	67.38	<0.001
PD whole tree	79.27 a	57.51 b	60.02 b	51.07 b	4.050	0.001
Shannon index	7.44 a	6.49 ab	6.95 ab	6.43 b	0.249	0.033
Simpson index	0.97	0.95	0.98	0.97	0.008	0.198

Different lowercase letters (“a” or “b”) within the same row indicate differences, “ab” indicates both similarities with “a” and “b”.

**Table 4:** Effect of the energy provision strategy on the rumen bacterial composition of Hu sheep at the level of phylum (>0.10%).

Phylum Name	LL	LH	HL	HH	SEM	*p*-Value
Bacteroidota	69.93	62.95	58.87	60.91	3.645	0.191
Firmicutes	25.39	33.48	34.55	35.93	3.301	0.138
Proteobacteria	1.82	0.80	1.56	1.64	0.418	0.351
Spirochaetota	0.44 b	0.86 a	0.64 ab	0.30 b	0.104	0.006
Verrucomicrobiota	0.77	0.43	0.76	0.25	0.184	0.154
Actinobacteriota	0.25	0.11	1.42	0.18	0.661	0.465
Desulfobacterota	0.35	0.43	0.42	0.33	0.124	0.916
Patescibacteria	0.17 ab	0.28 ab	0.47 a	0.08 b	0.091	0.035
Synergistota	0.31	0.17	0.40	0.08	0.093	0.098
Cyanobacteria	0.21	0.10	0.15	0.06	0.039	0.066

Different lowercase letters (“a” or “b”) within the same row indicate differences, “ab” indicates both similarities with “a” and “b”.

**Table 5:** Effect of the energy provision strategy on the rumen bacterial composition of Hu sheep at the level of genus (>0.50%).

Genus Name	LL	LH	HL	HH	SEM	*p*-Value
*Prevotella*	41.36	38.30	26.38	44.87	4.694	0.059
*Rikenellaceae RC9 gut group*	7.68 ab	10.08 ab	14.68 a	4.83 b	1.939	0.013
*Uncultured rumen bacterium*	6.48	4.79	6.20	4.78	1.066	0.553
*Christensenellaceae R-7 group*	3.55 b	2.26 b	11.38 a	4.39 b	1.513	0.002
*Uncultured bacterium*	5.46	5.87	6.80	2.59	1.257	0.134
*Prevotellaceae UCG-001*	3.94	4.27	2.88	3.05	1.570	0.904
*Selenomonas*	2.67 ab	3.57 ab	1.08 b	6.71 a	1.188	0.022
*Succiniclasticum*	2.78	3.10	3.72	4.17	0.875	0.683
*Prevotellaceae UCG-003*	5.76	1.64	3.43	2.55	1.211	0.128
*Veillonellaceae UCG-001*	2.01	1.61	2.25	1.66	0.416	0.670
*NK4 A214 group*	1.27	1.26	1.16	0.51	0.263	0.165
*Anaerovibrio*	0.51 ab	0.98 ab	0.15 b	2.49 a	0.401	0.003
*Ruminococcus*	0.76	0.76	0.52	1.21	0.165	0.055
*Butyrivibrio*	0.77	1.20	0.62	0.55	0.364	0.603
*Lachnospiraceae NK3A20 group*	0.42	0.79	1.10	0.83	0.374	0.645
*Anaeroplasma*	0.52 b	0.38 b	1.85 a	0.18 b	0.310	0.004
*Lachnospiraceae ND3007 group*	0.33	0.93	0.77	0.91	0.363	0.629
*Succinivibrio*	0.89	0.36	0.31	1.08	0.253	0.108
*UCG-002*	0.67	0.60	0.21	0.64	0.222	0.452
*Treponema*	0.39 b	0.84 a	0.57 ab	0.30 b	0.105	0.008

Different lowercase letters (“a” or “b”) within the same row indicate differences, “ab” indicates both similarities with “a” and “b”.

## Data Availability

The raw sequences generated from the twenty-four rumen fluid samples were deposited in the Sequence Read Archive (SRA) of the NCBI with the accession number PRJNA903143.
